# Transgene-host cell interactions mediate significant influences on the production, stability, and function of recombinant canine FVIII

**DOI:** 10.1038/mtm.2015.33

**Published:** 2015-11-18

**Authors:** Bredon Crawford, Margareth C Ozelo, Kenichi Ogiwara, James Ahlin, Silvia Albanez, Carol Hegadorn, Lori Harpell, Christine Hough, David Lillicrap

**Affiliations:** 1 Department of Pathology and Molecular Medicine, Queen’s University, Kingston, Ontario, Canada; 2 INCT do Sangue Hemocentro UNICAMP, INCT do Sangue Hemocentro UNICAMP, University of Campinas, Campinas, Brazil

## Abstract

Recombinant FVIII manufacturing is characterized by poor product stability and low yields. Codon-optimization of transgenes accelerates translation by exploiting the synonymous codon usage bias of a species. However, this can alter the performance of the final product. Additionally, the effects of transgene design across diverse cell types are not well understood and are of interest for next-generation protein and gene therapies. To investigate the effects of transgene design across different host cells, B-domain-deleted (BDD) and modified codon-optimized (CO-N6) transgenes were inserted via lentiviral delivery into cBOECs, HEK293T, and MDCK cells. The CO-N6 cFVIII transgene produced threefold more protein per transgene in HEK293T cells, and sixfold more protein in the two canine cell lines. However, pharmacokinetic analysis in hemophilia A dogs demonstrated that cFVIII produced from cBOECs transduced with the CO-N6 transgene had significantly reduced *in vivo* recovery. Furthermore, this product showed reduced *in vitro* stability and activity on thrombin activation versus the BDD product. This trend was reversed in HEK293T lines. Overall, our results demonstrate the need for an integrated approach that not only assesses protein expression levels but also considers the influence that host-cells have on preserving the molecular and biochemical properties of the naturally occurring FVIII.

## Introduction

Factor VIII (FVIII) is a plasma protein required for normal amplification of the intrinsic coagulation cascade and its deficiency causes hemophilia A. Severely affected patients are treated with either recombinant or plasma-derived FVIII. The pharmacokinetics and safety of these products are indistinguishable,^1^ and their availability has facilitated the implementation of regular prophylaxis as a therapeutic regime, resulting in better patient outcomes.^2,3^ However, production costs are high as a result of several biochemical characteristics of FVIII. These include inefficient expression of the FVIII mRNA,^4,5^ misfolding and degradation of the primary translation product,^6,7^ retention of the protein in the endoplasmic reticulum^8–11^ and the requirement for facilitated transport through the Golgi apparatus.^12^ Once secreted FVIII must be stabilized through interactions with von Willebrand factor (VWF).^13^

Initial efforts to improve production efficiency centered on enhancing the design of the FVIII cDNA. To reduce host cell metabolic load, the nonessential B domain was removed.^14^ However, although not necessary for procoagulant activity,^15^ evidence suggests that the B domain contributes to biosynthetic regulation by participating in quality control during protein synthesis and folding; shielding the protein from premature proteolysis by thrombin; reducing proteolysis by protein C and Factor Xa; and affecting clearance.^16^ The B domain contains N-linked glycosylation sites that interact with chaperones to facilitate transport through the endoplasmic reticulum. Reintroduction of a small portion of the B domain containing six N-linked glycosylation sites has significantly improved transport through the endoplasmic reticulum and enhanced the level of FVIII secretion.^17,18^

Low levels of FVIII expression have also plagued preclinical gene therapy trials and many of the modifications outlined above have been incorporated into various FVIII transgenes. Additionally, codon-optimization of the FVIII transgene has been investigated.^19–21^ Codon-optimization is a strategy in which synonymous codons are substituted for those that occur more frequently in highly expressed proteins. There is a direct correlation between these preferred codons and the relative abundance of corresponding tRNAs.^22^ However, codon usage bias is species-specific and it is thus somewhat surprising that despite adapting usage to a *Homo sapien* bias, optimized FVIII transgenes have been associated with supraphysiological levels of FVIII in hemophilia A mice.^19,21^ Codon-optimization of genes is extensively used in the production of many recombinant therapeutic proteins. Most of these proteins are produced in heterologous systems and optimizing human genes for these nonhuman expression systems has resulted in significant increases in expression.^23^ Although human recombinant FVIII is currently produced in heterologous systems, no analysis of the benefits of using a codon-optimized (CO) FVIII cDNA for recombinant production has ever been reported.

Host-cell engineering and culture optimization are as important as transgene design when optimizing large-scale productions of therapeutic proteins.^24^ The preference for mammalian expression systems derives from their ability to synthesize proteins that preserve molecular and biochemical properties present in the naturally occurring proteins. FVIII requires many post-translational modifications (PTMs) to produce a fully functional protein and therefore, the choice of host cell must be carefully considered since many of the enzymes required for correct PTM are species- and tissue-specific. Alterations to these natural modifications can influence immunogenicity^25^ and avoiding this is critical, since 30% of severe hemophilia A patients mount an immune response to their therapeutic protein. Therefore, to avoid immunogenic glycosylation patterns from murine cell lines, most recombinant FVIII is synthesized in hamster cells. Currently, investigations are underway to evaluate the use of immortalized human cell lines,^26^ but while these may eliminate possible species-mismatched glycosylation patterns, there is limited evidence that the currently manufactured recombinant FVIII products are more immunogenic than the natural FVIII isolated from plasma-derived products.^27^

The therapeutic significance of FVIII for hemophilia A patients, along with the technical challenges associated with production have consequently positioned FVIII as an important target in a number of emerging therapeutic protein technologies.^28,29^ Combinations of emerging technologies are hoped to provide synergistic improvements to recombinant FVIII productivity, stability, and half-life. However, the biological consequences of technology combinations are poorly characterized. With a view to increasing FVIII expression, we modified our canine (c) B domain-deleted (BDD) FVIII transgene that was used in an *ex vivo* gene delivery strategy^30^ and to produce recombinant FVIII for dogs in our hemophilia A colony. These modifications included codon optimization of the transgene and incorporation of a truncated region of the canine B domain containing six putative N-linked glycosylation sites (N6). In the present study, we investigated the effects of these transgene modifications when expressed in multiple host cell types, addressing cell line productivity, characteristics of the recombinant products, and effects on large animal pharmacokinetics.

## Results

### Codon optimization of the canine FVIII cDNA

**Figure 1:**
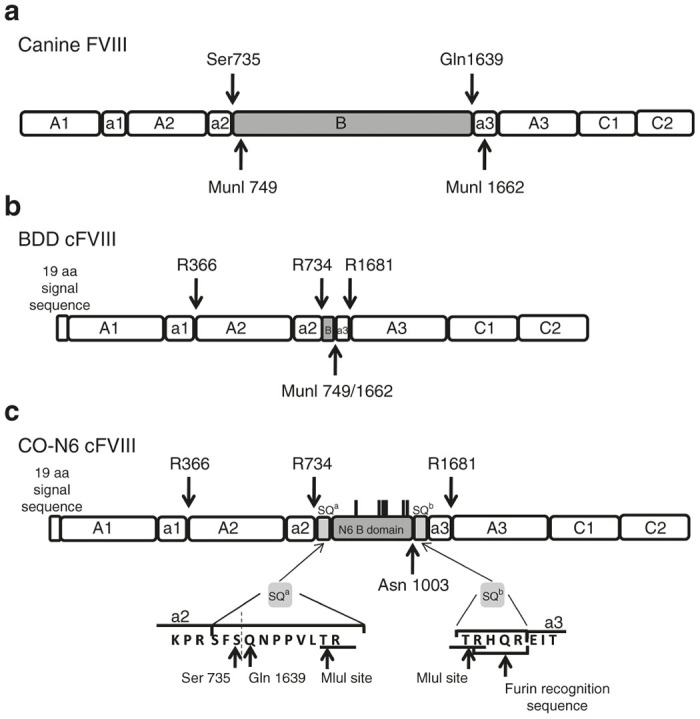
The cFVIII proteins produced with the B-domain-deleted (BDD) and CO-N6 cFVIII transgenes. (**a**) A cartoon representation of the wild-type cFVIII protein showing the domain structure and the boundaries of the B domain (Ser734–Gln1639). The MunI restriction sites located at amino acids 749 and 1662 were used to remove the B domain. (**b**) The protein encoded by the BDD cFVIII transgene showing the three thrombin cleavage sites at R366, R734, and R1681. A portion of the B domain (the first 15 amino acids) was retained while the rest of the B domain along with the first 15 amino acids of the a3 domain was removed. (**c**) The protein encoded by the CO-N6 cFVIII transgene contains the three thrombin cleavage sites. The B domain is bounded by two SQ sequences that contain MluI restriction endonuclease sites and a PACE/furin recognition sequence. The included B domain consists of the first 269 amino acids and contains six putative N-linked glycosylation sites at Asn857, Asn901, Asn1017, Asn1036, Asn1087, and Asn1099.

The nucleotide sequence of the cFVIII cDNA^31^ (Figure 1a) was used to generate the BDD cFVIII transgene (Figure 1b). This DNA sequence was used for codon optimization and includes the first 269 amino acids of the B domain that contains six putative glycosylation sites bounded by SQa and SQb^19^ linker sequences and is referred to as CO-N6 (Figure 1c). Both constructs have been incorporated into the lentiviral delivery system and are under the control of the ubiquitous EF1α promoter into which an endothelial enhancer element (-5.5HSCR) was added.^30^ A comparison of the CO-N6 cFVIII DNA sequence to the nonoptimized BDD sequence using Vector NTI software determined that optimization altered 1228 out of 5475 (23.75%) nucleotides. None of these changes altered any amino acids. DNA sequencing and analysis of the entire CO-N6 cFVIII transgene confirmed its integrity and ensured that it encodes a functional cFVIII protein.

### Cell lines and protein production

**Figure 2:**
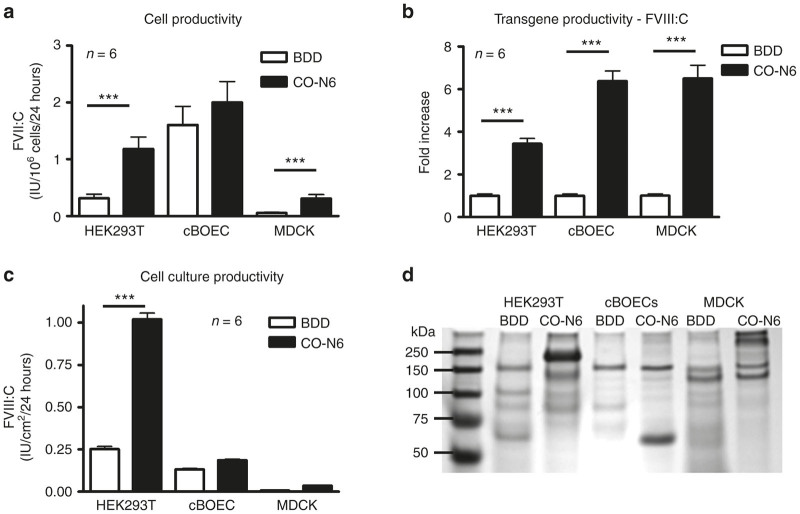
A comparison of recombinant cFVIII production from cBOECs, HEK293T, and MDCK cells transduced with either the B-domain-deleted (BDD) or CO-N6 cFVIII transgenes. (**a**) Levels of cFVIII:C produced from host cells transduced with either the BDD or CO-N6 cFVIII transgenes. cFVIII:C was measured after 24 hours in serum-free media and is expressed relative to 1 million cells. (**b**) The fold-increase in cFVIII:C production with the CO-N6 transgene. Transgene copy numbers were determined for each of the host cells transduced with either the BDD or CO-N6 transgenes and levels of cFVIII:C were determined relative to a single integrated copy of the transgene. The fold-increase in cFVIII production for the cells transduced with the CO-N6 transgene is expressed relative to the same cell type transduced with the BDD transgene. (**c**) Cell culture productivity is based on the surface area of the culture dish (cm^2^) and reflects differences in cell size, density and metabolism. All statistical comparisons between cells transduced with BDD or CO-N6 were made using an unpaired t-test. Error bars indicate standard deviation and (***) indicates *P* < 0.001, (**) *P* < 0.01, and (*) *P* < 0.05. (**d**) SDS-PAGE of the purified recombinant cFVIII products stained with Coomassie blue. Each of the purified recombinant cFVIII proteins was assessed for FVIII activity then diluted and loaded onto a SDS-PAGE gel. The expected molecular size of the single-chain cFVIII is approximately 160 kDa and the molecular weight of the additional 269 amino acids of B domain encoded by the CO-N6 cFVIII transgene is expected to be 29.68 kDa. A molecular weight marker is in lane 1 with associated molecular weights indicated to the left of each band. It should be noted that in order to visualize cFVIII on western blots it was necessary to load approximately twice as much purified protein onto the SDS-PAGE gel.

Three host cells, one human (HEK293T) and two canine (cBOECs and MDCK), were chosen for recombinant production of cFVIII. These cells were transduced with either Lenti-EF1α(–5.5HSCR)-cFVIII or Lenti-EF1α(–5.5HSCR)-CO-N6-cFVIII and serial dilutions of the transduced cells were cultured and expanded for at least 2 weeks. Cells expressing the highest levels of cFVIII:C were selected for further characterization. Levels of cFVIII:C were assessed in serum free media after culture for 24 hours and are based on 1 million cells (Figure 2a). While levels of cFVIII:C appeared to be higher in all cell-types expressing the CO-N6 cFVIII transgene, these levels are dependent on integrated transgene copy numbers and therefore transgene copy numbers for each cell-type were determined. Average integrated copy numbers for BDD-HEK293T and CO-N6-HEK293T cells were 0.95 or 1.04 copies/cell respectively, and 2.86 or 0.56 copies/cell respectively for BDD-cBOECs and CO-N6-BOECs. Transduction efficiencies were significantly lower with MDCK cells with only 5.6 × 10^−4^ and 6.8 × 10^−4^ integrated copies/cell in BDD-MDCK and CO-N6-MDCK cells. Transgene productivity, as represented by cFVIII:C per single integrated transgene, was determined and fold-increases for the CO-N6 cFVIII transgene relative to the BDD cFVIII transgene are shown in Figure 2b. cFVIII:C increased by 3.4-, 6.4-, and 6.5-fold in CO-N6-HEK293T, CO-N6-MDCK cells, and CO-N6-cBOECs, respectively. The increased expression from the CO-N6 transgene in canine cells was significant (*P* < 0.001) by a two-way analysis of variance and is consistent with optimization of the cFVIII transgene specifically for a canine codon bias.

Cell culture characteristics can directly affect recovered amounts of cFVIII and we therefore calculated total recovered cFVIII activity based on the surface area of the culture flask (Figure 2c) The higher cell densities associated with HEK293T cells made them overall more productive for cFVIII:C. cFVIII antigen (cFVIII:Ag) levels were measured in culture supernatants after 24 hours in serum-free media. Cell productivity for cFVIII:Ag was calculated relative to 1 million cells (Supplementary Figure 1a) and in contrast to cFVIII:C, levels of cFVIII:Ag from CO-N6-cBOECs were significantly less than that observed with BDD-BOECs. cFVIII:Ag productivity for a single integrated copy of the transgene and relative fold-increases in cFVIII:Ag expression were determined (Supplementary Figure 1b). Fold-increases in cFVIII:Ag from the CO-N6-HEK293T (2.9-fold) and CO-N6-MDCK (5.9-fold) cells were similar to the fold-increases in cFVIII:C (Figure 2b). However, only a twofold increase in cFVIII:Ag as compared to a 6.5-fold increase in cFVIII:C, was observed with CO-N6-BOECs.

Purified recombinant cFVIII from each of the three cell types transduced with either the BDD or CO-N6 transgene were assessed by SDS-PAGE (Figure 2d) and although there are similarities between the different isolated recombinant cFVIII proteins, each product has a distinct protein profile. Different PTMs in the three different host cell types, along with the increased size associated with inclusion of a portion of the B domain, and variable proteolysis, presumably account for the different banding patterns observed on the SDS-PAGE gel.

The apparent inefficient transduction and very low integrated transgene copy numbers in MDCK cells resulted in low levels of recombinant cFVIII production. Since significant amounts of cFVIII are required for pharmacokinetic evaluation, we chose to exclude these cells from further development. In addition, to facilitate treatment-scale production levels of cFVIII by increasing cell density, transduced cBOECs were seeded onto collagen-coated microbeads and maintained in stirred suspension culture. These cells tolerated elevated levels of shear stress, grew readily at high density and culture supernatant could be continuously recovered.

### Pharmacokinetic analysis

**Figure 3:**
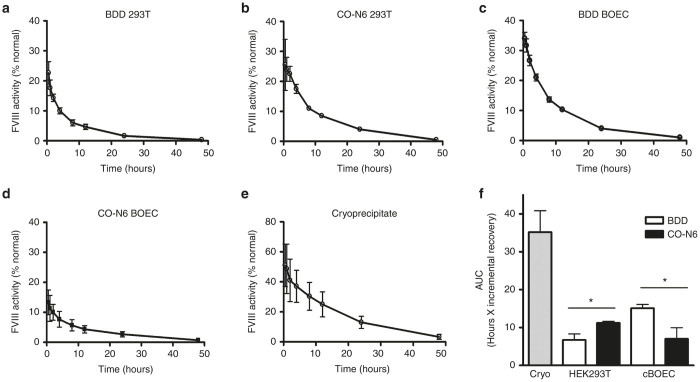
Pharmacokinetics of canine cryoprecipitate and recombinant cFVIII produced from cBOECs and HEK293T cells transduced with either the B-domain-deleted (BDD) or CO-N6 cFVIII transgenes (each infusion delivering 25 units/kg). (**a–e**) Profiles represent the mean FVIII activity over 48 hours. All recombinant cFVIII products were tested in the same three hemophilia A dogs except for the cFVIII that was isolated from CO-N6 cBOECs where safety concerns limited testing to only two dogs. Lines indicate best-fit two-phase exponential decay. (**f**) Bioavailability by area-under-the-curve. Canine cryoprecipitate (Cryo) was isolated from a normal dog. Error bars indicate standard deviation and (*) indicates statistical significance of *P* < 0.05.

Three hemophilia A dogs, two female and one male, each previously exposed to canine FVIII and weighing approximately 10 kg were selected for this study. None of these dogs had evidence of neutralizing anti-canine FVIII antibodies. Each dog received 25 units/kg of each of the recombinant cFVIII products via intravenous infusions over a 5-minute period of time. Blood samples were obtained 30 minutes 1, 2, 4, 8, 12, 24, and 48 hours later. Clearance was found to fit a two-phase exponential decay model for all recombinant cFVIII products as well as canine cryoprecipitate (Figure 3a–e). Notably, the second dog treated with cFVIII from CO-N6-HEK293T cells experienced reduced recovery and delayed peak activity. This was associated with a transient decreased heart rate, reduced blood pressure, pallor of the mucous membranes, and lethargy. These symptoms were observed again following a lower 10 U/kg test dose in the dog, and this product was suspended from additional *in vivo* testing (note that all cFVIII preparations were tested for endotoxin to ensure final endotoxin exposures of less than 0.2 EU/kg per dog). Notably, despite the tendency of some of the dogs in the colony to mount an anti-canine FVIII immune response^32^ none of the dogs treated with either the BDD or the CO-N6 products have developed inhibitory anti-canine FVIII antibodies. Area-under-the-curve (AUC) analysis using the trapezoidal rule (Figure 3f) indicated significant (*P* = 0.011) reduction in bioavailability of cFVIII from CO-N6-BOECs relative to cFVIII from BDD-BOECs. This contrasted with a significant (*P* = 0.032) increase in bioavailability of the cFVIII from CO-N6 HEK293T compared with cFVIII from BDD-HEK293T. Canine cryoprecipitate was prepared as control material and was found to have significantly (*P* = 0.016) higher bioavailability than all of the recombinant products tested. Reduced bioavailability was concomitant with low incremental recovery (Table 1). Canine cryoprecipitate showed a typical incremental recovery of 2.17 (U/dl)/(U/kg). This was significantly higher than all of the recombinant products tested (*P* = 0.015). cFVIII from CO-N6-BOECs had a significantly reduced recovery relative to the cFVIII isolated from BDD-BOECs (*P* = 0.008), consistent with AUC results.

*In vivo* procoagulant efficacy of each of the recombinant products was also assessed by determining whole-blood clotting times on samples taken from each to the time points (Supplementary Figure 2a–e). Whole-blood clotting times were compatible with the one-stage cFVIII:C assay results. Reduction in clot time was determined as the cumulative decrease in clotting time relative to baseline (Supplementary Figure 2f). These values were determined using the trapezoidal rule, analogous to AUC bioactivity described above.

### Stability and thrombin activation

**Figure 4:**
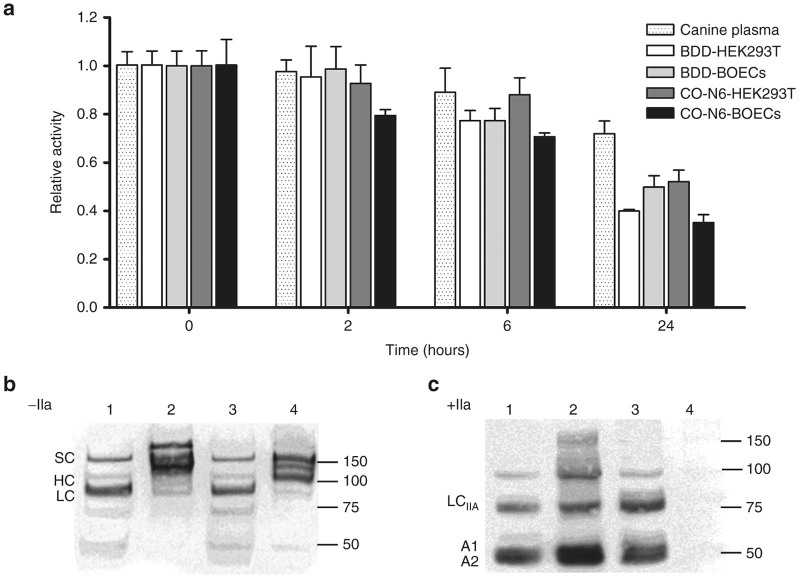
*In vitro* stability and thrombin digestion of recombinant cFVIII products. (**a**) To assess stability, the various recombinant cFVIII products produced in HEK293T or cBOECs transduced with either the B-domain-deleted (BDD) or CO-N6 transgenes were incubated at physiological concentration and temperature in citrated canine plasma. Remaining cFVIII:C was measured after 2, 6, and 24 hours and values are expressed relative to those observed prior to incubation (0 hours). Samples were incubated in triplicate and error bars indicate standard deviation. (**b,c**) Western blot analysis of recombinant cFVIII products incubated without (**b**) and with (**c**) thrombin. Molecular size markers are indicated to the right of each blot. Lanes 1 and 2 HEK293T cells; Lanes 3 and 4 cBOECs. Lanes 1 and 3, cells transduced with BDD transgene. Lanes 2 and 4, cells transduced with CO-N6 transgene. The bands for the single (SC), heavy (HC) and light (LC) chains of cFVIII, as well as the thrombin-activated light chain (LC_IIa_) and A1 and A2 domains are indicated.

To investigate factors that potentially affect pharmacokinetics, we assessed *in vitro* stability, levels of cFVIII activation by thrombin and decay of thrombin-activated cFVIII (FVIII_IIa_). Stability for the purified cFVIII was assessed by incubating each of the products at 37 °C for 2, 6, and 24 hours in citrated plasma isolated from hemophilia A dogs. cFVIII:C was calculated relative to initial values (Figure 4a). After 2 hours, >90% of the initial cFVIII:C was present in all of the products except for the cFVIII isolated from CO-N6-BOECs where only 79% of initial activity persisted. This loss in activity was significantly reduced (*P* = 0.023) when compared to the loss in cFVIII:C from the product produced in CO-N6-HEK293T. Significant reductions in activity were also observed at 6 hours (*P* = 0.01) and at 24 hours (*P* = 0.007) with the cFVIII produced in CO-N6-BOECs. The remaining cFVIII activity at 24 hours for all of the recombinant products was <50%, significantly less (*P* = 0.009) than the remaining cFVIII activity (72%) in canine plasma.

The recombinant cFVIII products were analyzed on western blots (Figure 4b) and while there are similarities between the size-distributions of BDD-encoded fragments, the banding patterns for CO-N6-encoded fragments are quite different and presumably represent different post-translational modification between the two cell-types. The predicted sizes for thrombin-cleaved A1 (50 kDa), A2 (43 kDa), and A3/C1/C2 were observed for products derived from BDD-HEK293T, CO-N6-BOECs, and CO-N6-HEK293T cells (Figure 4c). However, the anti-canine FVIII antibody did not detect any thrombin-cleavage products associated with the cFVIII isolated from CO-N6-BOECs, even when a 10-fold increase in the amount of cFVIII was used (data not shown).

**Figure 5:**
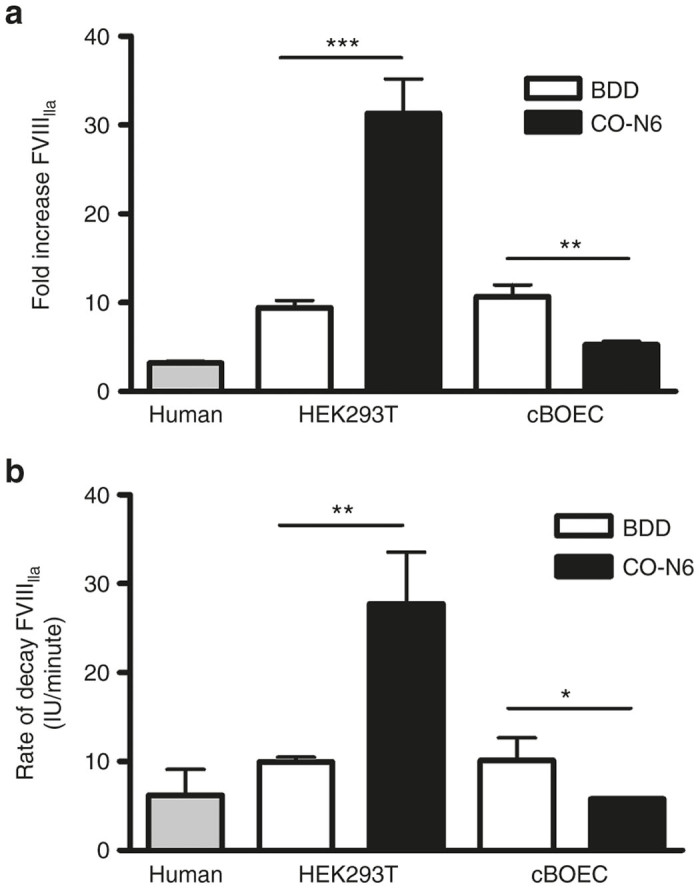
Thrombin activation. Recombinant cFVIII products were incubated with or without thrombin for 1 or 30 minutes and levels of cFVIII activity were measured using an aPTT-based assay. (**a**) The fold increase in levels of cFVIII_IIa_ after 1 minute incubation with thrombin relative to initial levels of unactivated cFVIII. (**b**) The rate of decay of the various activated recombinant cFVIII products. *N* = 3 for all experiments. Error bars indicate standard deviation and (***) indicates *P* < 0.001, (**) *P* < 0.01, and (*) *P* < 0.05.

The presence of contaminating activated cFVIII in a purified preparation can result in an overestimation of recovered recombinant cFVIII. Although analysis of nonactivated recombinant products (Figure 2d and Figure 4b) suggests little or no contaminating activated cFVIII, we proceeded to confirm this. Purified products were incubated for 30 minutes and cFVIII activity, relative to initial values, was determined. No rapid loss of activity, associated with contaminating activated cFVIII was observed (data not shown). Thrombin-catalyzed activation of cFVIII (cFVIII_IIa_) and rates of decay of cFVIII_IIa_ were examined (Figure 5). The fold-increases in activity after thrombin activation for both cFVIII products produced in BDD-HEK293T and BDD-BOECs are quite similar (~10-fold) (Figure 5a). However, this was not the case for the recombinant cFVIII products produced in CO-N6-HEK293T and CO-N6-BOECs. There was a significant difference (*P* = 0.0003) between the fold-increase of cFVIII_IIa_ when CO-N6-HEK293T (5.3-fold) and CO-N6-BOECs (31-fold) were used to produce the cFVIII. We also evaluated the rates of decay of cFVIII_IIa_ (Figure 5b) and once again the rates were quite similar with the recombinant cFVIIIs encoded by the BDD transgene but were significantly different (*P* = 0.0028) with the recombinant cFVIIIs encoded by the CO-N6 transgene. All the cFVIII products had a greater fold-increase in levels of thrombin-activation and rate of decay than that observed for recombinant human FVIII.

## Discussion

Recombinant production of FVIII is complicated by inherent low levels of production and the requirement for PTMs to produce a fully functional protein. The need for PTMs limits production of FVIII to mammalian cells and currently, most commercially available recombinant FVIII products are produced in either CHO or BHK cells. While these heterologous cells can carry out PTMs, concern has been expressed that subtle differences between the two cell types might contribute to observed variable risks of inhibitor development between products.^27,33,34^

We wanted to evaluate the use of a modified cFVIII transgene as a way to increase levels of protein production, and to assess whether or not different host cells influence the levels of production, integrity, and function of the purified recombinant products. We used a lentiviral delivery system to genetically modify cells, since this gene-integrating platform simplifies evaluation and comparisons of cFVIII production from two different cFVIII transgenes expressed in various host cells. Finally, pharmacokinetic analysis of each of the products was carried out in the same three hemophilia A dogs, thereby eliminating confounding variables such as age, weight, and other genetic modifiers of pharmacokinetic behavior. While it would appear that use of a CO transgene increases cFVIII production, it is important to note that there are other differences between this transgene and the original BDD construct that could also account for some of this observed benefit. First and foremost, the modified transgene includes an additional 269 amino acids from the canine FVIII B domain, 6 of which are putative N-linked glycosylation sites that have been shown to facilitate FVIII secretion. In addition, it also contains an optimized Kozak sequence to enhance translational efficiency. In contrast, the BDD transgene only contains 15 amino acids from the B domain, none of which are putative glycosylation sites. Since the cFVIII transgene was codon optimized for a canine bias, it is not altogether surprising that levels of cFVIII expression were higher when the modified transgene was expressed in either cBOECs or MDCK cells. However, host cell characteristics also affected levels of production. Specifically, HEK293T cells are smaller and can grow at a much higher density, and these features almost certainly contribute to the higher levels of cFVIII produced from both BDD-HEK293T and CO-N6-HEK293T cells. Notably, levels of cFVIII are higher from BDD-HEK293T cells than CO-N6-BOECs and CO-N6-MDCK cells. Production of recombinant canine FVIII was previously described in baby hamster kidney (BHK) cells stably transfected with a BDD FVIII cDNA.^35^ This cDNA contains virtually none of the BDD^36^ and yet when expressed in BHK cells is associated with higher yields and increased stability relative to a similar human BDD construct.

The strategy to modify the original BDD FVIII transgene and to use cBOECs was developed with a view to produce an ideal therapeutic by balancing improved transgene design with the most native-like cellular processing available. BOECs can be readily isolated, transduced to express FVIII and expanded to the numbers required for either recombinant protein production or to provide a therapeutic benefit in gene delivery strategies. Furthermore, endothelial cells are normally exposed to shear stress, and we used this biomechanical influence to enhance levels of cFVIII production by seeding transduced cBOECs on collagen-coated microbeads and culturing them in spinner flasks. This allowed for extensive expansion of cell numbers and facilitated prolonged and continuous harvesting of increased levels of the recombinant cFVIII. In addition, we previously demonstrated efficacy with these cells in an *ex vivo* gene delivery strategy in murine^37^ and canine^30^ models of hemophilia A. We have not yet evaluated the CO-N6 transgene in our *ex vivo* delivery strategy in hemophilia A dogs as the initial trial with the BDD FVIII transgene was associated with an anti-FVIII immune response. This is an adverse event that has been reported previously following FVIII gene therapy and is likely not due primarily to the use of cBOECs. However, there are also limitations associated with the use of BOECs, in particular when these cells are transduced to express FVIII. Despite the fact that endothelial cells are the native cells for synthesis of FVIII, and they also produce the VWF that protects FVIII from premature inactivation and clearance, we have previously found that cBOEC levels of VWF drop rapidly once these cells are genetically modified to express high levels of FVIII.^30^ Furthermore, we have also observed that elevated levels of cFVIII expression in transduced cBOECs are associated with increased cBOEC apoptosis.^30^

Pharmacokinetic profiles showed that cFVIII derived from CO-N6-cBOECs had poor bioavailability and *in vitro* evaluations showed compromised cFVIII stability and thrombin activation. Since codon optimization was not the only modification that was made to the CO-N6 transgene, it is not possible to definitively conclude that the diminished functional activity of this protein is a direct consequence of codon-optimization. However, codon-optimized proteins have been shown to exhibit altered function and increased susceptibility to protein degradation.^35,38–40^ The loss of rare codons has been shown to cause functional alterations to proteins^38,41^ as they are thought to contribute to pauses during translation, thereby increasing the probability of achieving thermodynamically-favored cotranslational folded-states.^39^ Thus, while the combination of the canine CO-N6 transgene expressed in native canine cells is associated with increased transgene expression, concordant with more efficient protein translation, it may also be associated with aberrant secondary or tertiary protein structure. There are several observations that support the presence of aberrantly folded cFVIII species in the purified preparation from CO-N6-cBOECs. First, the polyclonal antibody used to measure cFVIII antigen for the enzyme-linked immunosorbent assays as well as the western blot, appears to have a much lower affinity for the cFVIII produced in CO-N6-cBOECs. Specifically, while there is good correlation between the fold-increases of cFVIII:C and cFVIII:Ag with the CO-N6-HEK293T and CO-N6-MDCK cells, this correlation does not persist with CO-N6-cBOECs, and significantly less than expected cFVIII antigen is detected (we do not expect that the cFVIII from CO-N6-cBOECs has an enhanced specific activity to explain this altered ratio). Furthermore, thrombin activation experiments and long-term incubation confirmed altered function and stability when combining canine codon-optimization with the canine translational apparatus, consistent with structural alterations to the protein. However, the most striking evidence of aberrant folding in the codon-optimized proteins was the loss of epitopes on the recombinant cFVIII produced in CO-N6-cBOECs following high-concentration thrombin incubation. This suggests that the material had increased susceptibility to promiscuous thrombin digestion,^40^ or that the digested products were antigenically dissimilar to native cFVIII and the other products tested. These aberrantly folded forms of cFVIII may have persisted into the final products in the absence of affinity purification, and may explain the very low cFVIII activity observed. When infused intravenously, active but misfolded forms of the recombinant cFVIII might be quickly cleared, resulting in significantly reduced recovery levels 30 minutes after injection.

A subset of the design features in the CO-N6 transgene, such as the reincorporation of a truncated B-domain,^17^ may be beneficial to recombinant cFVIII product function. The glycosylation profile of cFVIII facilitates secretion and is also essential to ensure structural stability and biological activity. Thromboelastographic studies showed that the CO-N6-HEK293T and CO-N6-BOEC derived cFVIII products produced significantly higher α angle values (a descriptor of clot growth rate—not shown) than BDD-HEK293T and BDD-BOECs derived cFVIII. Additionally, the terminal half-life of cFVIII from CO-N6-HEK293T and CO-N6-BOECs approached that of cryoprecipitate. When the remaining activity at 24 hours is considered relative to peak activity, the cFVIII from CO-N6-cBOECs significantly outperforms cFVIII from BDD-HEK293T and BDD-BOECs (not shown), despite having reduced recovery. The deletion of the B domain in the cFVIII BDD transgene removes 24 amino acids of the acidic a3 domain. This region (amino acids 1664–1681) is involved in VWF binding. In contrast, it has previously been shown that deletion of the FVIII B domain does not affect binding to VWF.^42^ Although the VWF binding region and the critical Tyr1673 are not removed in our BDD construct they are now directly juxtaposed with the proximal region of the remaining B domain and this may affect the affinity of the BDD product for VWF. In products isolated from HEK293T cells, cFVIII activity was comparable, however the CO-N6 product had a greater AUC suggesting a genuinely longer half-life. It is noteworthy that we included a putative furin cleavage site in the CO-N6 transgene as it was thought to be critical for intracellular processing and cleavage of FVIII. However, it has very recently been show that furin cleavage actually hampers the biological activity of FVIII.^43^ By every metric tested, the cFVIII from CO-N6-HEK293T was superior to the BDD version, demonstrating both the potential of this transgene and the significant role of species- and cell-type specific processing of this recombinant protein. The important role of rare codons in directing folding pathways provides opportunities to further improve transgene design. For example, a map of wild-type gene translation speeds could be adapted to the codon bias to improve folding accuracy. Additionally, recent kinetic modelling suggests that fast codons may also play a regulatory role by rushing the protein through functionally useful but thermodynamically unfavored conformations.^44^ By preserving slow regions and making fast regions faster, it may be possible to develop designs that increase both protein yield and quality.

In conclusion, the results that we provide here show that a family of products, developed in parallel can provide clues to inform transgene design considerations for recombinant FVIII production and/or gene therapy.

## Materials and Methods

### Generation of the BDD and codon-optimized canine FVIII transgenes

The canine (c) FVIII cDNA^31^ (Figure 1a) was used to generate Lenti-EF1α(–5.5HSCR)-cFVIII. This construct contains a BDD cFVIII transgene under the control of the ubiquitous EF1α promoter into which an endothelial enhancer element was added.^30^ MunI sites at amino acids (AAs) 749 and 1,663 were used to remove the B domain while retaining the thrombin cleavage sites at AA 734 and 1681 (BDD cFVIII: Figure 1b). This DNA sequence was used for codon optimization and additionally includes the first 269 amino acids of the B domain that contains six putative glycosylation sites bounded by SQa and SQb^19^ linker sequences (CO-N6 cFVIII: Figure 1c). The nucleotide sequences directly 5’ to the ATG start of translation were modified to introduce a Kozak consensus sequence and an additional TGA stop codon was added to the 3’ end of the gene. XhoI and XmaI restriction sites were added to the 5’ and 3’ ends of the CO-N6 cFVIII sequence to facilitate cloning into the lentiviral plasmid. The CO-N6 cFVIII cDNA was generated for a canine codon usage bias with GeneOptimizer, a proprietary software program developed by GeneArt and was manufactured by GeneArt (Invitrogen, Carlsbad, CA) and cloned into the Lenti-EF1α(–5.5HSCR)-cFVIII, thereby replacing the BDD cFVIII transgene to generate Lenti-EF1α(–5.5HSCR)-CO-N6-cFVIII.

### Genetic modification of host cells to express cFVIII

Lentiviral vectors were produced as described previously.^37^ Briefly, HEK293FT cells (Invitrogen) were cotransfected with pMDLgag/pol RRE, pRSV-Rev, pCI-VSV-G, and either Lenti-EF1α-(–5.5HSCR)-cFVIII or Lenti-EF1α-(–5.5HSCR)-CO-cFVIII constructs via calcium phosphate precipitation with 7, 7, 13, and 40 μg respectively of each plasmid. Viral particles were isolated from conditioned media by ultracentrifugation at 20,000 rpm for 2.5 hours using a Beckman SW28 rotor (Beckman Coulter Mississauga, Canada), resuspended in 20 mmol/l Tris-HCl with 2 mmol/l MgCl_2_ and 5% sucrose and stored at −80 °C. Functional titres were determined as previously outlined.^37^

cBOECs were isolated as previously described.^37^ Briefly, buffy coat mononuclear cells isolated from canine venous blood were seeded in MCDB131 (Invitrogen) supplemented with 2 mmol/l L-glutamine, 100 IU/ml penicillin, 100 μg/ml, streptomycin, 10% fetal bovine serum, and EGM-2 SingleQuot (Lonza, Mississauga, Canada) to a 12-well plate coated with rat tail type-I collagen (BD Biosciences, Mississauga, Canada). Colonies with endothelial morphology formed within 2–3 weeks, becoming confluent approximately 10 days later. MDCK cells (ATCC; cat. CCL-34) and HEK293T cells were cultured in Dulbecco’s Modified Eagle’s medium high glucose (Corning, Tewksbury, MA) with 10% fetal bovine serum. cBOEC, HEK 293T, and MDCK cells were transduced at multiplicities of infection between 5 and 250 using a Viraductin Lentivirus Transduction kit (Cell Biolabs, San Diego, CA) and expanded. To determine levels of cFVIII expression, type-I collagen-coated six-well plates were seeded overnight with 10^6^ cells per well for each combination of multiplicities of infection, transgene, and parental cell type, rinsed in Hank’s buffered salt solution and incubated for 24 hours in serum-free expression media. Expression media used for MDCK cells was MDCKUltra medium (Lonza), and for HEK293T cells it was Dulbecco’s Modified Eagle’s medium/F12 without phenol red medium supplemented with 2 mmol/l L-glutamine, 2.5 mmol/l CaCl_2_, insulin-transferrin-selenium, and 1 mg/ml Albumax (Invitrogen). cBOEC expression media comprised endothelial basal medium without phenol red (Lonza) supplemented with EGM-2 (excluding fetal bovine serum), 100 IU/ml penicillin, 100 μg/ml streptomycin, 2.5 mmol/l CaCl_2_ insulin-transferrin-selenium, and 1 mg/ml Albumax. FVIII activity in expression media was measured with a one-stage assay on an STA compact hemostasis system (Stago) from three on-board dilutions against a standard curve of normal canine pooled plasma. Plates were then trypsinized and cells were counted in a hemocytometer. The most productive cell lines were expanded and stored.

### Production and purification of recombinant cFVIII

For large scale-productions, HEK 293T cells were cultured in 500 cm^2^ triple T-flasks (Sigma-Aldrich, Oakville, Canada), using the expansion and expression media described above and harvested daily for 2–3 days. cBOEC were expanded in static culture to 5 ×·10^7^ cells and subsequently seeded onto 5 g of Cytodex 3 collagen-coated microbeads (Sigma-Aldrich) prepared according to the manufacturer’s instructions. Cells were expanded to confluency in a 1 l spinner flask (Bellco Glass, Vineland, NJ), using the serum-free medium described above. Confluent beads were washed three times in Hank’s buffered salt solution, and resuspended in the BOEC serum-free expression medium described above and harvested daily for 2–3 days. Conditioned expression media was supplemented with 10 μm Amidophenylmethanesulfonyl fluoride and 1 mmol/l benzamidine, then passed through a 0.45 μm Steritop vacuum filter (Millipore, Etobicoke, Canada). Filtrate was purified as described previously^35^ with minor modification. Briefly, filtrate was loaded onto a 50 ml SP-Sepharose Fast Flow column (GE Healthcare, Mississauga, Canada) equilibrated with 20 mmol/l 2-(*N*-morpholino)ethanesulfonic acid (MES), 0.15 M NaCl, 5 mmol/l CaCl_2_, 0.01% Tween-80 (Sigma-Aldrich), pH 6.8. The column was washed with buffer and eluted 20 mmol/l MES, 0.65 M NaCl, 5 mmol/l CaCl_2_. Following one-stage clotting assay, fractions with cFVIII activity were stored at −80 °C. Fractions from successive daily harvests were thawed, pooled, and diluted with 20 mmol/l 4-(2-hydroxyethyl)-1-piperazineethanesulfonic acid (HEPES), 5 mmol/l CaCl_2_, pH 7.4. The diluate was loaded on 5 ml Q-Sepharose Fast Flow column (GE Healthcare) equilibrated with the same buffer. The column was washed with buffer and eluted with 20 mmol/l HEPES, 5 mmol/l CaCl_2_, 0.65 M NaCl pH 7.4. Fractions were supplemented with 3% sucrose and stored at −80 °C in small aliquots. Products were assayed for endotoxin using a chromogenic Limulus Amebocyte Lysate assay (Thermo Scientific, Mississauga, Canada), to ensure final exposures of less than 0.2 EU/kg per dog. Lot concentrations were determined by diluting the products 1:100, 1:200, and 1:400 in severe hemophilic dog plasma prior to one-stage clotting assay as described above. HEK293T, cBOECs, and MDCK host cells transduced with the BDD transgene are referred to as BDD-HEK293T, BDD-BOECs, BDD-MDCK respectively, while the same cells transduced with the CO-N6 transgene are referred to as CO-N6-HEK293T, CO-N6-BOECs, CO-N6-MDCK respectively.

### Functional cFVIII and antigen assays

Levels of cFVIII activity (cFVIII:C) were measured with a one-stage activated partial thromboplastin time-based assay using a STA compact hemostasis system (Stago, Toronto, Canada) from three on-board dilutions against a standard curve of normal canine pooled plasma. When necessary, cFVIII samples were diluted in canine deficient plasma. cFVIII and cVWF antigens were quantified by enzyme-linked immunosorbent assay as previously described^30^ using a polyclonal sheep anti-cFVIII antibody (SAC8C-IG; Affinity Biologicals, Ancaster, Canada) and a polyclonal rabbit anti-human VWF antibody (Dako, Mississauga, Canada) that cross reacts with canine VWF antigen (Dako). Normal canine pooled plasma was used to generate the standard curve. The anti-cFVIII antibody used in our canine FVIII enzyme-linked immunosorbent assay was generated against cFVIII produced from our BDD cFVIII transgene product and should therefore not interact with the B domain of the full-length native FVIII in the pooled plasma.

### Animals

The previously described^32,45^ hemophilia A dog colony is housed at Queen’s University in facilities that are accredited by the Canadian Council for Animal Care. All procedures were approved by the Queen’s University Animal Care Committee.

### Pharmacokinetics

Production of normal canine cryoprecipitate and measurement of whole-blood clotting times were completed according to standard protocols. Following a 2-week washout period, three dogs were each treated with 25 U/kg of FVIII, derived from cryoprecipitate and each of the recombinant cFVIII products. Blood samples were obtained via the saphenous vein. Parametric pharmacokinetic analysis was modeled with simple one-phase-exponential decay. Nonparametric area-under-the-curve was calculated using the trapezoidal rule.^46^ Curve-fitting, two-way analysis of variance, and *t*-tests were conducted with Prism 4 (GraphPad, La Jolla, CA).

### Activation of recombinant FVIII with thrombin

cFVIII samples were diluted to 0.3–0.5 U/ml in 20 mmol/l HEPES, 150 mmol/l NaCl, 4 mmol/l CaCl_2_, 10 g/l bovine serum albumin, pH 7.4 in a final volume of 200 μl. To each sample, 1 μl of a 1.2 U/ml Recothrom (Bayer, Mississauga, Canada) solution was added, vortexed, and incubated in a water bath at 37 °C for 1 or 30 minutes. Volumes of 20 μl were removed, 6 μl of 36 U/ml hirudin (ProspecBio, East Brunswick, NJ) were added and after vortexing, samples were diluted 10-fold to ensure negligible amounts of hirudin were carried into the one-stage activated partial thromboplastin time-based assay.^47^ Samples were immediately assayed for cFVIII:C. Controls were incubated without thrombin and were used to calculate levels of activation of cFVIII by thrombin.

### Stability of recombinant cFVIII

To evaluate stability, aliquots of the purified recombinant cFVIII were diluted 1:100 in canine hemophilic plasma, incubated at 37 °C in a water bath for 2, 6, or 24 hours and cFVIII:C was immediately assessed.

### SDS-PAGE and western blot analysis

The various recombinant cFVIII proteins were diluted to approximately 10 μg in deionized water and incubated at 37 °C for 30 minutes with dithiothreitol and 3× SDS sample loading buffer (New England Biolabs, Whitby, Canada). Proteins were separated on a 10% acrylamide Next Gel (AMRESCO, Solon, OH), and electroblotted in running buffer without methanol to a polyvinylidene fluoride membrane with 0.2 μm pore size in a Mini-Protean three-cell electrophoresis system (Bio-Rad, Mississauga, Canada). Membranes were blocked with 5% skim milk powder in Tris-buffered saline and probed with a peroxidase-conjugated sheep anti-canine FVIII antibody (Affinity Biologicals). Following washing with 0.1% Tween-20 in Tris-buffered saline, membranes were rinsed in Tris-buffered saline then Western Lightning ECL chemiluminescent substrate (PerkinElmer, Woodbridge, Canada), and imaged in a FluorChem IS-8900 imaging system (Alpha Innotech, San Leandro, CA) over a 15-minute exposure. For thrombin digestion of cFVIII, 5 μl samples were preincubated for 15 minutes in 1.2 IU Recothrom.

## Figures and Tables

**Table 1:** Pharmacokinetic analysis from three hemophilia A dogs treated with recombinant cFVIII derived from different host cells transduced with either the BDD or CO-N6 transgenes

	*Cryoprecipitate*	*BDD HEK293T*	*CO-N6 HEK293T*	*BDD cBOECs*	*CO-N6 cBOECs*
Mean incremental recovery (U/dl)/(U/kg)	2.17 (1.93–2.42)	0.90 (0.62–1.19)	0.96 (0.55–1.38)	1.42 (1.23–1.62)	0.56 (0.27–0.85)
Terminal half-Life (hours)	13.91	6.50	13.76	9.19	14.34

BDD, B-domain-deleted.
