# Supplementary figures and images for: Transgene-host cell interactions mediate significant influences on the production, stability, and function of recombinant canine FVIII
Source: Mol Ther Methods Clin Dev. 2015 Nov 18;2:15033–. doi: 10.1038/mtm.2015.33 (PMC4650998; doi:10.1038/mtm.2015.33)

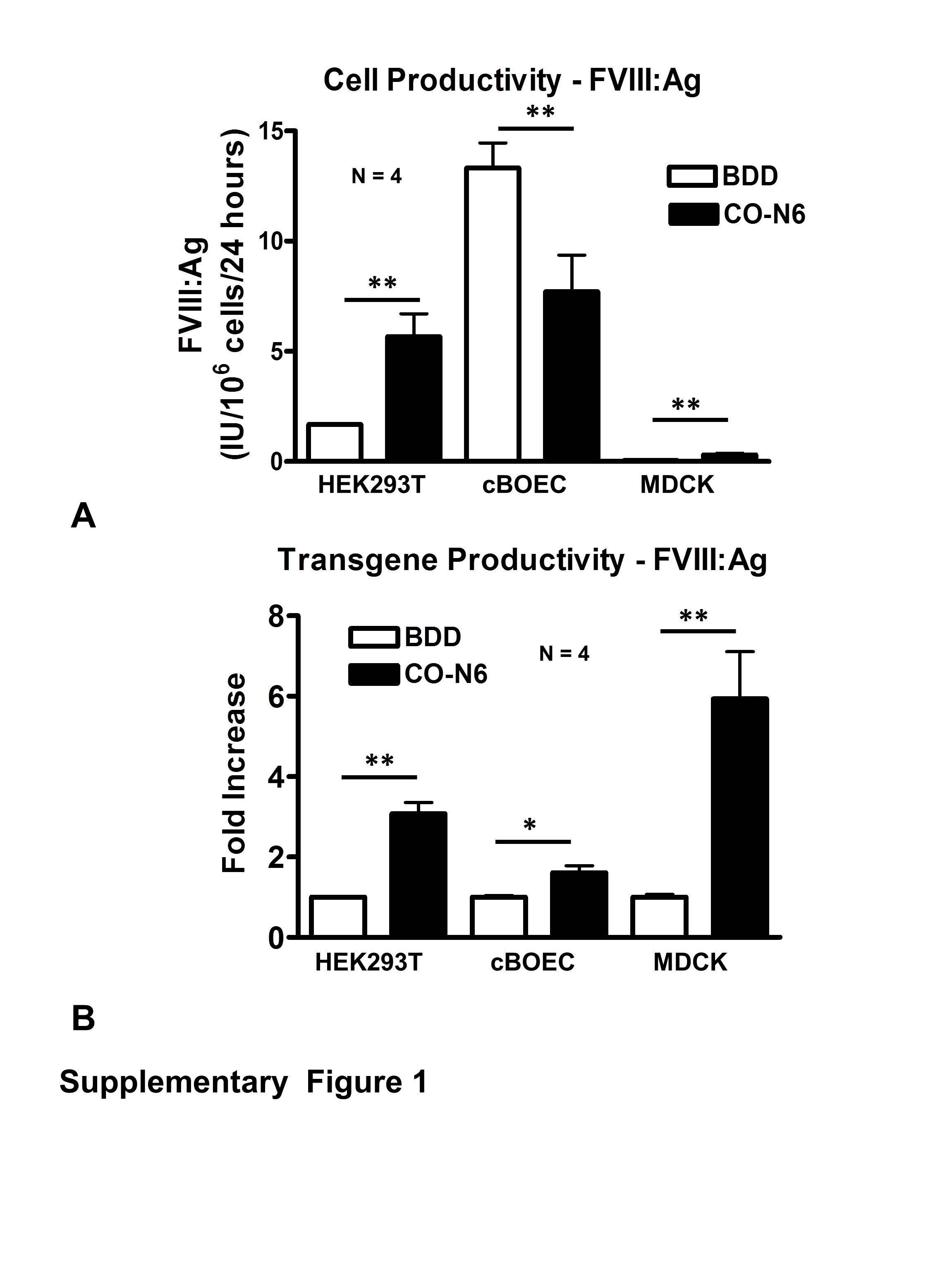

Supplement: Supplementary Figure S1 [file mtm201533-s1.tiff]

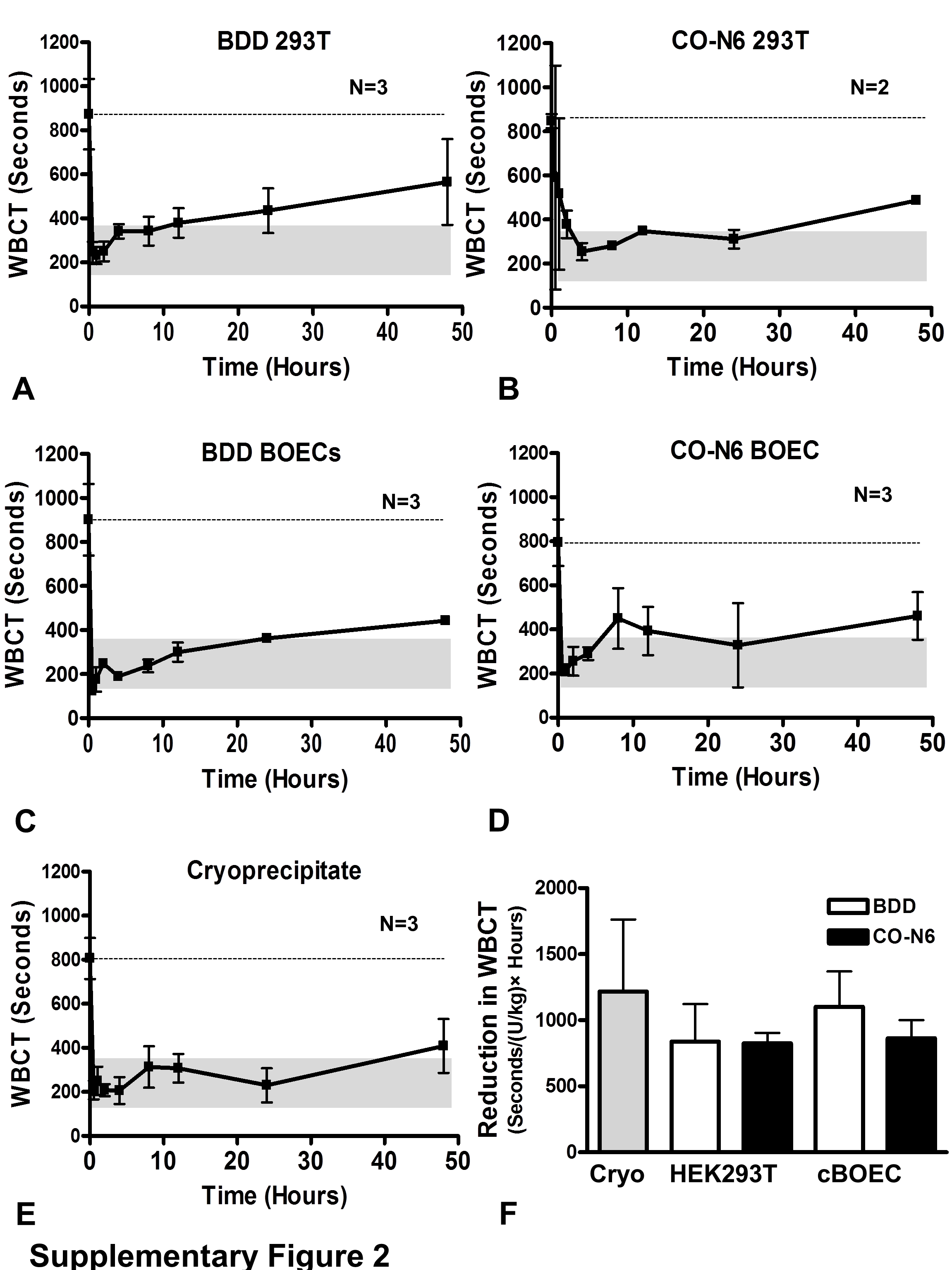

Supplement: Supplementary Figure S2 [file mtm201533-s2.tiff]
